# Novel insights into osteocyte and inter-organ/tissue crosstalk

**DOI:** 10.3389/fendo.2023.1308408

**Published:** 2024-01-17

**Authors:** Yan Zhang, Qingchang Chen

**Affiliations:** ^1^Department of Pediatrics, Union Hospital, Tongji Medical College, Huazhong University of Science and Technology, Wuhan, China; ^2^Department of Ultrasound Medicine, Union Hospital, Tongji Medical College, Huazhong University of Science and Technology, Wuhan, China; ^3^Clinical Research Center for Medical Imaging in Hubei Province, Wuhan, China; ^4^Hubei Province Key Laboratory of Molecular Imaging, Wuhan, China

**Keywords:** osteocyte, osteocyte-organ communication, brain, kidney, vascular calcification

## Abstract

Osteocyte, a cell type living within the mineralized bone matrix and connected to each other by means of numerous dendrites, appears to play a major role in body homeostasis. Benefiting from the maturation of osteocyte extraction and culture technique, many cross-sectional studies have been conducted as a subject of intense research in recent years, illustrating the osteocyte–organ/tissue communication not only mechanically but also biochemically. The present review comprehensively evaluates the new research work on the possible crosstalk between osteocyte and closely situated or remote vital organs/tissues. We aim to bring together recent key advances and discuss the mutual effect of osteocyte and brain, kidney, vascular calcification, muscle, liver, adipose tissue, and tumor metastasis and elucidate the therapeutic potential of osteocyte.

## Introduction

When the term “postmenopausal osteoporosis” was first pointed out by Fuller Albright in the 1940s, awareness to its clinical features, pathogenesis, and pathophysiology has gradually increased (1). Evidence from numerous studies revealed that bone was a dynamic tissue and played a crucial role in muscle attachment and structure support together with a mineral reservoir such as phosphate and calcium, which are critical to normal physiological function. In 2000, a great research carried out by Ducy et al. demonstrated the brain–bone connection through the effect of leptin on bone metabolism (2), leading to an explosion of studies on bone and other organs’ crosstalk.

Known as progenitor cell reservoir, more than 12 types of cell lineages arising from hematopoietic and mesenchymal stem cells existed in the skeletal system or released to blood circulation (3). Among them, the most prevalent and longest-living terminally differentiated cells found in bone tissue are osteocytes (4, 5). They act as mechanosensory units, with OCY forming a network similar to the morphology and connectedness of the neural system (6). Owing to the difficulty of obtaining cells that lay in the solid mineralized matrix, the understanding of OCY biology was delayed and unilateral. Over the past few decades, growing experimental evidence described a mature OCY extraction technique by a series of collagenase digestions and calcium chelation, providing opportunity to specialize osteocytic molecular biology and function (7, 8).

**Figure 1:**
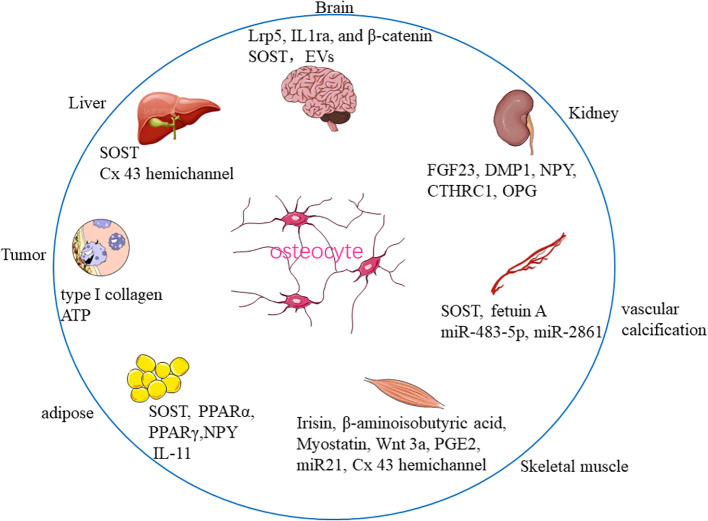
Communication between osteocyte and distant organs/tissues.

Recent studies further reported that bone tissue communicated not only with closely situated organs, for instance, bone marrow, skeletal muscle, and fat tissue, but also with vital organs outside the skeleton, such as the kidney, liver, and brain, indicating that the skeletal system may be an elaborate and sophisticated organ (9). In this review, we sought to provide the most recent evidence on the crosstalk between bone-residing OCY and other organs/tissues, namely, brain, kidney, vascular calcification, muscle, liver, adipose tissue, and tumor metastasis to elucidate the therapeutic prospect of OCY (**Figure 1**).

## OCY in physiologic conditions

Apart from undergoing programmed cell apoptosis, approximately 5%–20% of matrix-producing osteoblast progressively transitioned to terminal OCY accompanied by distinct functional and morphological changes (10). Osteoblast is cuboid-shaped, with well-developed endoplasmic reticulum cells mostly located on the surface of bone tissue. After being embedded in a mineralized matrix, cells were surrounded by collagen, with cell volume reduced by approximately 70%, and with extensive arborization, endoplasmic reticulum and mitochondria reduction, and dendrite extension forming a neuronal-like morphology, making OCY actively connected to each other (10). In addition, genetic and epigenetic reprogramming such as cytosine DNA methylation is also tightly correlated with OCY differentiation and maturation (11). For instance, sclerostin (SOST, an osteocytic specific glycoprotein) expression was modulated by DNA methylation during osteoblast–osteocyte transition (12). In postmenopausal women, increased SOST promoter methylation resulted in lower serum SOST level and stimulated bone formation by inhibition of Wnt signaling activity (13). During the OCY differentiation process, H3K27me3 in the loci of osteocyte-expressing genes decreased and H3K27me3 demethylase was attached to those genes (14). Osteocytogenesis is also accompanied by increased expression of dentin matrix protein 1 (DMP1), matrix metalloproteinases (MMPs), and fibroblast growth factor 23 (FGF23), which is critical for OCY maturation, dendritic formation, and elongation together with phosphate metabolism (15, 16).

Acting as primary skeletal mechanosensors, OCY sense mechanical signals by changes in interstitial fluid flow shear stress (FFSS) travel over their dendrites to initiate bone remodeling (17). OCY have been shown to regulate bone remodeling progress through osteoblastic anabolic factor nitric oxide (NO), prostaglandin E2 (PGE2), and osteoclast differentiation regulator receptor activator of nuclear factor-κ B ligand (RANKL) secretion (4, 18–21). Furthermore, it also exhibited capability to reorganize new mineral on the perilacunar/canalicular matrix. Disturbed osteocyte-driven perilacunar remodeling resulted in OCY osteolysis in pathologic conditions such as hyperparathyroidism, hypophosphatemic rickets, and osteoporosis (22). Moreover, the crosstalk between OCY and other organs has become a hot topic in the past few decades. Within much less cytoplasm and/or organelles, OCY were considered a silent placeholder that resides in the solid matrix. However, recent studies identified that OCY were a major source of many potent local and soluble cytokines that contribute to osteoblast and osteoclast differentiation and function (23, 24). OCY-derived molecules/cytokines have proven to be effective mediators in the communication between OCY and other organs or tissues (**Table 1**).

**Table 1:** Molecules/cytokines that participated in osteocyte and inter-organ/tissue crosstalk.

	Molecules/cytokines	Physiological function
OCY and brain	Lrp5, IL1ra, and β-catenin	Tumor-suppressing capability in brain metastases (25)
	SOST	Reduce social hierarchy (26)
	Evs contain Aβ degradation and mitochondrial energy metabolism factors	Ameliorate neuronal cell apoptosis and cognitive impairment (27)
OCY and kidney	DMP1	Minimize the consequence of adverse cardiovascular outcomes in CKD (28)
	FGF23	Increase renal Pi excretion and reduce 1,25(OH)2D production (29)
	NPY	Renoprotective effects (30) or faster CKD progression (31)
	CTHRC1 and OPG in hUSC-EVs	Anti-osteoporotic effect (32)
OCY and VC	SOST	Correlate with epigastric and coronary artery calcification extent (33), time-dependent with VC (34)
	miR-483-5p and miR-2861	Exacerbate large calcium deposition lesion areas (35)
	ASHG	Anti-calcification activity (36)
OCY and skeletal muscle	Irisin	Prevent OCY apoptosis and increase SOST production (37, 38)
	β-aminoisobutyric acid	Prevent reactive oxygen species-inducted OCY cell death (39)
	Mbtps1 protease	Stimulate muscle regeneration and muscle contractility (40)
	Myostatin	Stimulate the expression of SOST and RANKL (41)
	Wnt 3a	Promote myogenic differentiation and enhance contractile force (42)
	PGE2	Stimulate primary myoblast proliferation (43)
	miR21	Influence skeletal muscle size in a sex-dimorphic manner (44)
	Cx43 hemichannel	Reinforce fast-twitch skeletal muscle mass (45, 46)
OCY and liver	SOST	Correlate with liver dysfunction (47, 48)
	Cx43 hemichannel	Cx 43-positive OCY existed in alcoholic liver cirrhosis (49)
OCY and adipose tissue	SOST	OCY ablation leads to low adipose tissue mass (50, 51)
	PPARα, PPARγ	BMAT accumulation, adipose browning and fat infiltration (52, 53)
	NPY	Stimulate adipogenic differentiation (7)
	IL-11	Inhibit adipogenesis (23)
OCY and tumor	Type I collagen	Tumor shrinkage effect (54)
	ATP	Suppress breast cancer cell migration (55)

## Brain–OCY crosstalk

In the past decades, considerable evidence identified that bone is richly innervated by nerve fibers and alters metabolic and anabolic activities to systemic and local factors (7, 56). Histological staining further revealed nerve distribution most often in a metabolically active area by identifying enzymes and neuropeptides (57, 58). Among all the cells that reside in the bone tissue, OCY is the most special one. Similar to neurons, OCY presents a large amount of cilia and cytoplasmic processes (≈40–100 per cell) surrounded by mineralized bone matrix to connect with adjacent cells (27), leading researchers to question the connection between OCY and neuron cells.

In the regulation from brain to OCY, the hypothalamus and the pituitary gland are the main segments that participate and release neurohormones. Limited by the difficulty of OCY extraction and culture technology, plenty of investigations showed that nearly all neurohormones had an effect on osteoblasts or osteoclasts (but not OCY) and exhibited bone anabolic or catabolic roles to regulate skeletal integrity; thus, the central control of OCY activity is almost blank (59–62). A recent study showed that the brain-derived neurotrophic factor, a growth factor mostly originated from the nervous system, plays a positive role on the proliferation of the murine osteocytic cell line MLO-Y4 (63). Co-injection of OCY into the brain exhibited tumor-suppressing capability through Lrp5, IL1ra, and β-catenin upregulation in brain metastases that occur from advanced breast cancer (25). Intracerebroventricular injection of SOST regulated social–emotional reactions such as anxiety-like behaviors, together with reduced social hierarchy and dendritic complexity of pyramidal neurons in mouse hippocampus (26).

In addition to communicating via soluble molecules, extracellular vesicles (EVs) and their specific cellular materials are an intriguing subject matter (64). EVs are cell-derived membranous structures containing proteins, lipids, and genetic material that exchange biological signal from cell to cell. According to their differing size, biogenesis, and membrane protein profile, EVs were characterized with exosomes, microvesicles, and apoptotic bodies. After being released from the original cell, EVs flow into the blood system and selectively merge with a target cell to elicit biological responses by vesicle content (65, 66). Jiang et al. isolated osteocytic extracellular vehicles (OCY-EVs) from 2‐ or 16‐month‐old mice, respectively. EVs isolated from young-aged mice played a protective role in β‐amyloid peptide pathology and neuronal cell apoptosis, and ameliorated cognitive impairment in an Alzheimer’s disease mouse model. Proteomic quantitative analysis defined more than 310 proteins highly enriched in young mice OCY-EVs; among them, Aβ degradation and functional factors of mitochondrial energy metabolism mainly participated in cognitive impairment and pathogenesis of Alzheimer’s disease, indicating a novel mechanism in bone–brain communication (27). EVs together with neurohormones mediated the crosstalk between brain and OCY, which needs to be fully depicted.

## OCY and kidney

Skeleton was recognized as a static organ that only provides muscle attachment and structure support to the body movement until the observation of FGF23, a growth factor secreted by OCY regulating phosphate homeostasis in the kidney, leading investigators to think of bone tissue as an endocrine gland. Stagnant osteoblast and OCY maturation are characteristic features of chronic kidney disease (CKD) bone (67). In clinical work, renal patients are often observed with bone disease such as osteoporosis and osteomalacia owing to significant derangements of electrolyte metabolism (68, 69), suggesting the possibility that kidney and bone communicate reciprocally.

Dendritic-shaped OCY is a major source of circulating signaling factors such as FGF23, DMP1, neuropeptide Y (NPY), and SOST. Recent studies suggested that OCY altered the production of several hormones critically involved in mineral metabolism in a very early stage during CKD, reflecting alterations in OCY metabolism (70). In detail, DMP1 minimized the consequence of adverse cardiovascular outcomes in CKD by preventing OCY apoptosis and FGF23 elevation, indicating a protective role of DMP1 in CKD patients (28). In addition, DMP1 is a negative regulator of FGF23 transcription. FGF23 acts on kidney and parathyroid glands to increase renal Pi excretion and reduce 1,25-dihydroxyvitamin D (1,25(OH)2D) production, resulting in phosphate waste and hypophosphatemia. OCY ablation markedly increased intestinal Pi absorption and stimulated renal Pi excretion (29). Furthermore, NPY is a 36-amino-acid peptide mostly produced by the nervous system and OCY (7). In acute kidney injury (AKI) mouse models, NPY exhibited renoprotective effects through Y1R by blocking M1 macrophage activation and renal necroinflammation (30). However, another cohort study pointed out that NPY was associated with proteinuria, faster CKD progression, and higher risk of kidney failure (31).

New research indicated that human urine-derived stem cell-derived EVs (hUSC-EVs) play a crucial role in abnormal bone metabolism. hUSC-EVs exerted an anti-osteoporotic effect through stimulating bone formation and suppressing bone resorption by transferring collagen triple-helix repeat containing 1 (CTHRC1) and osteoprotegerin (OPG) (32). Owing to imbalanced mineral homeostasis, CKD exacerbated cortical and trabecular bone loss and microarchitectural degradation associated with aging (71). Thus, it is likely that OCY and kidney closely communicate with each other despite the relatively long distances.

## OCY and vascular calcification

Vascular calcification (VC) is a complex and highly regulated process and often related to cardiovascular complications (72). Calcium phosphate deposition, especially hydroxyl calcium phosphate, is the main step of VC (73). VC most often co-exists with bone disorder in the early stage of CKD patients, resulting in increased morbidity and mortality together with poor outcome in a prematurely aged patient population (74, 75). Though a specific mechanism for this syndrome is yet to be revealed, OCY secretion stimulation seems to take part in this process.

Among all the OCY-derived cytokines involved in VC progress, SOST is the most studied one. Evaluated by coronary artery computed tomography, circulating SOST level was positively correlated with epigastric and coronary artery calcification extent and may be a predictor of vascular calcification (33). Triggered by renal malfunction, local osteocytic production of 1,25(OH)2D was increased, protecting the organism from ectopic calcification by increasing SOST and suppressing BMP2 production in early CKD patients (76). Warfarin-exposed rats developed a time-dependent VC along with a continuous increase in SOST levels; this process was mainly achieved through inhibiting Wnt/β-catenin signaling and inducing PPARγ signaling (34).

Using a VD3-induced acute VC mouse model, Wang et al. reported that EVs from aged bone matrix exacerbate large calcium deposition lesion areas in abdominal aortas. Meanwhile, the same phenotype was observed in chronic VC experiment models. Owing to the special localization of OCY in bone matrix, together with miRNA array analysis, they speculated that aged OCY demonstrate a positive role on VC by transferring miR-483-5p and miR-2861 (35).

A new study reported that fetuin A (ASHG), a circulating glycoprotein with anti-calcification activity, was mostly produced by OCY consistently. This production process was modulated by FGF23 (36). ASHG-deficient mice exhibited soft tissue calcification changes such as myocardium, lung, pancreas, kidney, and the skin, leading to delayed growth and premature death (77). Paradoxically, another study illustrated that circulating ASHG level has no correlation with VC in hemodialysis patients, while age, diabetes mellitus, and parathyroid hormone (PTH) levels were independent predictors for these patients (78).

## OCY crosstalk with skeletal muscle

Skeletal muscle and bone are the two mechanically loading tissues in the musculoskeletal system affecting each other through mechanical interaction and in an endocrine and paracrine manner. They often displayed tissue mass synchronization throughout our whole life. Exercise maintains increased muscle mass and bone mineral density (39). Disuse or lack of physical activity led to muscle atrophy and eventually resulted in OCY apoptosis due to hyposecretion of multiple hormone-like molecules (79).

Mechanical loading derived by muscle contraction plays a pivotal role in skeletal health. Transient muscle atrophy induced by local injection of botulinum toxin led to muscle inactivity and immobilization, resulting in increased vascular canal porosity, diminished OCY lacunar density, and terminal osteoporosis (80, 81). Moreover, molecular coupling of muscle and bone was established in recent years. Specific muscle-derived factors exerted a critical part in OCY response to loading by activating PI3K/Akt and β-catenin signaling pathways (82). Irisin, a myokine secreted by skeletal muscle, prevented disuse-induced OCY apoptosis and increased both osteocytic survival and SOST production in a direct manner (37, 38). Furthermore, exercise-induced muscle factor β-aminoisobutyric acid played a bone-protective role under oxidative stress by preventing reactive oxygen species-inducted OCY cell death (39). Conditional deletion of osteocytic Mbtps1 protease stimulated muscle regeneration and reinforced muscle contractility with age by upregulation of *Pax7*, *Myog*, *Myod1*, *Notch*, and *Myh3* gene expression (40). Myostatin, a myokine that negatively regulates muscle growth, was found to directly affect OCY and indirectly influence other bone cells. Myostatin markedly stimulated the expression of SOST, DKK1, and RANKL while inhibiting miR-218 expression in cultured osteocytic (Ocy454) cells, and negatively regulated osteoblastic differentiation in an indirect manner, indicating a novel mechanism in muscle–bone crosstalk (41).

OCY secretome also accounts for various molecules and miRNAs that affect skeletal muscle. After being secreted by OCY, soluble factors such as Wnt 3a exhibited a positive effect on myogenic differentiation, enhancing contractile force and calcium release (42). OCY released large amounts of PGE2 signaling on G1-S phase cell cycle progression to stimulate primary myoblast proliferation (43). OCY-derived miR21 influenced skeletal muscle size in a sex-dimorphic manner. In detail, female mice were susceptible while male mice seemed unaffected (44). Furthermore, partial ablation of DMP1-positive OCY caused severe sarcopenia, osteoporosis, and degenerative kyphosis, leading to shorter lifespan in these animals.

In addition to osteokines, a recent study showed that the osteocytic connexin (Cx) 43 channel plays a crucial role in bone–muscle crosstalk and PGE2 partially engaged in this process. Deletion of Cx43 reduced fast-twitch skeletal muscle mass together with protein synthesis and increased protein degradation (45). In aged mice, Cx43 hemichannel impairment displayed a protective role on bone mass while compromising skeletal muscle function due to increased muscle collagen deposition (46).

## Liver–OCY communication

Acting as a nutrient and energy metabolic center, the communication between liver and other solid organs has been deeply recognized. However, much less study was conducted about the relationship between liver and bone, especially OCY, even though OCY-mediated skeletal health is tightly correlated with liver disease (83). Clinical data identified that almost all patients who suffered from chronic liver diseases associate with altered bone metabolism, particularly severe osteoporosis, leading to a novel research area named hepatic osteodystrophy (84).

Researchers reported that resident liver stem cell (RLSC) spontaneously differentiated to OCY after cultivation in osteogenic condition for half a month, suggesting an OCY differentiative potentiality of RLSC (85). For cirrhotic patients, fewer OCY existed while serum SOST level was significantly elevated and clearly correlated with liver dysfunction markers such as albumin (47, 48). A significant decline in OCY lacunar feature was detected in lumbar vertebrae with female alcohol-associated liver disease (86). A recent study further showed that the severity of liver tissue disturbances was associated with impaired functionality and defected signal transduction of OCY lacunar network. In detail, fewer Cx 43-positive OCY were detected in vertebral and femoral bone in alcoholic liver cirrhosis individuals from 40 cadaveric men (49). Research work conducted in this field is very limited; future studies are required to fully verify the relationship between OCY and liver.

## Osteocyte and adipose tissue

Age- and menopause-related skeletal disturbances are closely associated with imbalanced bone remodeling characterized by decreased bone formation, increased osteocyte apoptosis, and bone marrow adipose tissue (BMAT) accumulation. Emerging evidence suggests that OCY and adipose tissue mutually influence each other in a direct and indirect manner. Sato et al. reported that ablation of osteocyte lead to severe lymphopenia and complete loss of white adipose tissues (87). In addition, global deletion of SOST exhibited dramatic increases in bone mass, together with low adipose tissue mass and impaired insulin sensitivity (50). Subcutaneous adipose tissue SOST was reduced after sprint interval training (51). Since SOST is almost produced by OCY, these findings prompted researchers to think about the intricate interaction between osteocyte and fat metabolism (87).

Peroxisome proliferator-activated receptors (PPARs) represent a group of fatty acid-activated transcription factors that regulate energy metabolism (52). Recent studies identified that PPARα and PPARγ were essential factors for the connection between OCY and adipose tissue. PPARα is expressed in OCY and plays a vital role in controlling bone marrow adiposity together with peripheral fat metabolism. OCY-specific deletion of PPARα led to BMAT accumulation and beginning of inguinal white adipose tissue, with no effect on bone mass or microarchitecture (52). In addition, PPARγ deletion in OCY resulted in upregulated adipose browning and decreased fat infiltration in skeletal muscle and liver (53). By means of constructing an osteocyte-specific lack of NPY mouse model, Zhang et al. revealed an osteocyte NPY‐dependent neuronal control of bone marrow mesenchymal stem/stromal cells’ differentiation fate. In detail, OCY secrete excess NPY to stimulate adipogenic differentiation instead of osteogenic differentiation through cAMP/PKA/CREB signaling during aging and osteoporosis (7). A recent study identified interleukin-11 (IL-11) as a mediator of bone-adipose crosstalk in a mechanical loading process. Osteoblast/osteocyte-specific IL-11 deletion mice exhibited blunted bone formation and increased systemic adiposity. A mechanism study further clarified that IL-11 directly inhibited adipogenesis to enhance Wnt signaling by suppressing Dkk1 and 2 (23). In contrast, BMAT accelerated bone deterioration through palmitate-mediated lipotoxicity on OCY, including induced apoptosis and reduced autophagy (88).

Because they are easily harvested and used for autologous implantation, adipose-derived mesenchymal stromal cells (ASCs) have become a hot topic in regenerative medicine and tissue engineering for the treatment of cartilage and bone disorders. ASCs are multipotent and can differentiate into various cell types such as OCY, chondrocytes, and myocytes (89). Exosomes isolated from ASCs effectively inhibited OCY apoptosis and OCY-mediated osteoclastogenesis through suppressing reactive oxygen species production and mitochondria-dependent signal activation (90). Experimental and clinical applications are needed to further explore the therapeutic potential of ASC in an OCY-related disease.

## Tumor–OCY interaction

Primary bone tumors are relatively rare, but because of the highly vascularized and metastatic environment (TGFβ-rich calcified matrix), bone tissue is the third most common site of solid tumor metastasis, with up to 70% of metastatic breast and prostate cancer patients harboring bone metastasis, leading to shortened survival time and serious bone complications during the remaining lifespan (91–94). Distant metastasis such as bone tissue is considered one of the primary causes of treatment failure in advanced breast cancer (5, 55). Among all the bone cells, OCY seems less affected by metastasizing cancer cells because they are further away from the actively metabolized bone marrow. However, recent studies illustrated that OCY played an integral role in tumor metastasis in an endocrine and non-endocrine manner.

Matrix-laden OCY and their cultured medium inhibited the proliferation, migration, and progression of mammary tumor cells both *in vivo* and *in vitro*. OCY coinjection further reduced tumor-driven osteolysis and achieved tumor-suppressive activity through an Lrp5-mediated Wnt signaling pathway (95). In return, mechanically loaded breast cancer cells modulated cell growth, OCY mechanosensing, and dendrite formation (96). OCY also mediated a tumor shrinkage effect through type I collagen, the major organic component within the bone. Furthermore, migratory breast cancer cells were attracted by OCY through bone matrix protein (54). Extracellular ATP released by osteocytic connexin hemichannels suppresses breast cancer cell migration and bone metastasis (55).

By using a prostate cancer metastasis cell line, DU145, Santen et al. illustrated that conditioned medium extract from shear-loaded OCY differentially altered epithelial and mesenchymal gene expression and decreased prostate cancer invasion while having no effect on cell proliferation (97). Paradoxically, another study using a co-culture, organ-chip model demonstrated inhibition of metastatic breast and prostate tumor growth while increasing cell invasion with mechanical stimulation of OCY (98). Other non-metastatic bone cancers such as adenocarcinoma, ovarian cancer, and Lewis lung carcinoma displayed increased OCY lacunar area and OCY death, in line with empty lacunae (99).

Multiple myeloma (MM) is a malignancy of the plasma cells, characterized by osteolytic destruction, which exhibited tumor expansion preferentially and bone-destructive lesions (100, 101). Triggered by activation of Notch signaling, OCY underwent caspase-3-dependent apoptosis and increased osteocytic RANKL and SOST expression in MM (102). Inhibition of SOST protein significantly increased bone formation, decreased fracture susceptibility, and thus prevented the development of bone disease in MM patients (100). Apoptosis, together with OCY autophagy, might also be involved in OCY–MM interaction (103). OCY apoptosis was dramatically increased in bone areas colonized by MM cells, and autophagic death was triggered after being cocultured with MM cells (103, 104).

## OCY as treatment targets

Bisphosphonates (BPs) have long been used to preserve bone mass through an antiresorptive effect by inhibiting bone-resorbing osteoclast activity (105, 106). The pharmacological effects of BPs depend on bone mineral affinity and inhibitory effects depend on biochemical targets to bone cells, especially farnesyl diphosphate synthase, which plays a vital role in the bone resorption process (107). Benefiting from the lacuna-canalicular network, BPs can travel a certain distance and bind with OCY lacunae to exert antiapoptotic effect on OCY strictly dependent on Cx43 expression but not gap junctions (108–110). BPs with lower affinity seem to penetrate deeper into the canalicular network than higher-affinity compounds for the bone mineral (110). Denosumab is an antibody against RANKL, a cytokine mostly produced by OCY, which exhibited potently anti-resorptive and anti-fracture properties. However, persisting lower OCY viability and elevated fracture risk was identified after denosumab discontinuation (111), resulting in OCY apoptosis and multiple vertebral osteonecrosis (112). The monoclonal antibody anti-SOST, romosozumab, displayed a dual effect on bone metabolism by stimulating bone formation and inhibiting resorption. In postmenopausal osteoporosis patients, romosozumab treatment evidently decreased vertebral fracture risk at 12 months and after the transition to denosumab at 24 months (113). Proteasome inhibitor treatment increased OCY viability and blunted dexamethasone-induced OCY death through autophagy modulation in MM patients (103). Parathyroid hormone exerted pro-resorptive skeletal effects through activating osteocytic Notch signals and SOST expression downregulation (114). Research on the therapeutic potential of OCY is far from sufficient; additional efforts are warranted to explore potential future therapies.

## Conclusion and perspectives

Embedded in the solid bone matrix, OCY was thought of as a silenced cell, a placeholder in bone, not having contact with other tissue. Past research mostly focused on the role of bone-formation osteoblast and bone-resorption osteoclast; within the last decade, considerable studies of OCY have strikingly increased, resulting in the discovery of novel functions of OCY. Even though the crosstalk between bone and other organ has been widely studied, the relationship between OCY and neighbor or distant tissues is not yet fully elucidated.

Because the world population’s life expectancy continues to increase, aging-related diseases such as brain dysfunction and bone loss represent a major public health problem. Over the past decades, researchers have described many aspects of osteoporosis and neurodegenerative disease, which is strongly correlated with clinical epidemiology, but there remain many unanswered questions. The interplay between brain and OCY under normal and pathological conditions needs to be fully elucidated.

In this review, we summarized new insights into the mutual influence of OCY and other organs, presenting the therapeutic potential of OCY, which has become a target of intervention in related diseases. Further study in this field is needed to achieve a better understanding of the underlying mechanisms of manifestations in the bone, and vice versa, and provide new pathways for research on body homeostasis.

## Author contributions

YZ: Funding acquisition, Writing – original draft. QCC: Funding acquisition, Writing – review & editing.
